# Imaging plasma formation in isolated nanoparticles with ultrafast resonant scattering

**DOI:** 10.1063/4.0000006

**Published:** 2020-06-18

**Authors:** Daniela Rupp, Leonie Flückiger, Marcus Adolph, Alessandro Colombo, Tais Gorkhover, Marion Harmand, Maria Krikunova, Jan Philippe Müller, Tim Oelze, Yevheniy Ovcharenko, Maria Richter, Mario Sauppe, Sebastian Schorb, Rolf Treusch, David Wolter, Christoph Bostedt, Thomas Möller

**Affiliations:** 1 LFKP, ETH Zürich, 8093 Zürich, Switzerland; 2 Max-Born-Institut Berlin, 12489 Berlin, Germany; 3 IOAP, Technische Universität Berlin, 10623 Berlin, Germany; 4 ARC, La Trobe University, 3086 Melbourne, Australia; 5 Stanford PULSE Institute, SLAC National Laboratory, Menlo Park, California 94305, USA; 6 FLASH at DESY, 22607 Hamburg, Germany; 7 Institut de Minéralogie, de Physique des Matériaux, et de Cosmochimie (IMPMC), Sorbonne Université, UMR CNRS 7590, Muséum National d’Histoire Naturelle, 75005 Paris, France; 8 ELI Beamlines, Institute of Physics, Czech Academy of Science, 252 41 Dolní Břežany, Czech Republic; 9 European XFEL, 22869 Schenefeld, Germany; 10 German Aerospace Center (DLR) Berlin, 12489 Berlin, Germany; 11 Paul-Scherrer Institute, CH-5232 Villigen PSI, Switzerland; 12 LUXS Laboratory for Ultrafast X-ray Sciences, Institute of Chemical Sciences and Engineering, École Polytechnique Fédérale de Lausanne (EPFL), CH-1015 Lausanne, Switzerland

## Abstract

We have recorded the diffraction patterns from individual xenon clusters irradiated with intense extreme ultraviolet pulses to investigate the influence of light-induced electronic changes on the scattering response. The clusters were irradiated with short wavelength pulses in the wavelength regime of different 4d inner-shell resonances of neutral and ionic xenon, resulting in distinctly different optical properties from areas in the clusters with lower or higher charge states. The data show the emergence of a transient structure with a spatial extension of tens of nanometers within the otherwise homogeneous sample. Simulations indicate that ionization and nanoplasma formation result in a light-induced outer shell in the cluster with a strongly altered refractive index. The presented resonant scattering approach enables imaging of ultrafast electron dynamics on their natural timescale.

## I. INTRODUCTION

Intense femtosecond short-wavelength pulses from free-electron lasers (FELs) open new avenues to investigate transient states and ultrafast processes with unprecedented spatial and temporal resolution.^1–4^ One prominent example is ultrafast x-ray diffraction methods such as femtosecond Coherent Diffraction Imaging (CDI), which have enabled the structure determination of individual nonperiodic nanoscale objects.^5^ The elastically scattered photons of a single-shot exposure form an interference pattern containing a snapshot of the object before it is quickly destroyed due to the large amount of deposited energy.^6,7^ The encoded structural information can be retrieved via phase retrieval methods^8^ or forward simulations,^9^ which allowed for the structural characterization of such fragile objects as single viruses,^10,11^ aerosols,^12,13^ atomic clusters,^9,14,15^ and even superfluid helium nanodroplets containing quantum vortices.^16,17^ Via pump-probe techniques, laser-induced processes in individual nanoparticles can also be studied in a time-resolved manner with unprecedented spatiotemporal resolution.^18–22^ Such studies are pivotal for understanding and mitigating the damage dynamics from ionization, plasma formation, and particle explosion, which limit the achievable resolution in CDI experiments.^7,23^

While it may be possible to outrun the structural damage induced in the nanoscale targets,^6^ the ultrafast changes in the electronic structure due to excitation, ionization, and plasma formation occur on a faster, few-femtosecond or subfemtosecond timescale, unseparable from the interaction with the intense pulse.^24–27^ The sensitivity of the diffraction process to the particle's electronic structure, on the other hand, holds tremendous opportunities to trace electronic structure changes with high spatial resolution in a time-resolved manner with ultrafast diffraction methods.^28^ In particular, near absorption resonances, the x-ray scattering cross sections depend sensitively on the energy of the incoming photons and the electronic structure of the target.^27–30^

In this work, we demonstrate that ultrafast resonant scattering can be used to visualize the spatial distribution of transient charge states in an evolving nanoplasma. As samples, we use submicrometer-sized xenon clusters that are transformed to a highly excited nanoplasma during irradiation and imaged with the same intense femtosecond FEL pulse. On the timescale of the pulse, the position of the clusters is frozen in space, and the measured ion kinetic energies show that ionic motion in the generated nanoplasma can be neglected. Nevertheless, in the radial intensity profiles of single-shot single-cluster scattering patterns, we find an intensity dependent lobe structure corresponding to the appearance of an additional characteristic length in the otherwise homogeneous particle. At the FEL wavelength used in our study, neutral xenon and also its low charge state ions (Xe^≤4+^) are strongly absorbing, while higher charge states are almost transparent. The choice of a resonant wavelength allows us to discriminate between areas of different charge states. We carried out a one-dimensional Monte Carlo simulation of the photoionization process, suggesting the formation of a highly charged outer shell in the evolving nanoplasma with strongly altered optical properties. By grouping and averaging the experimental patterns obtained at similar FEL intensities, we suppress individual cluster effects, e.g. from a rough surface, while enhancing the relevant dynamic signature. The radial profiles of the grouped patterns are fitted with Mie-calculations for a concentric core–shell model to extract tendencies of the evolution as a function of illumination intensity. The fitting yields a sequence of core–shell structures with strongly altered refractive indices and increasing shell thickness. The experiments demonstrate the possibility to extract spatial information on transient plasma formation in isolated nanoparticles with resonant ultrafast x-ray scattering. The method provides the potential for imaging ultrafast excitation, ionization, and charge transfer dynamics in complex samples with femtosecond time and nanometer spatial resolution.

## II. EXPERIMENT

The experiments were performed at beamline BL2 at the soft x-ray free-electron laser FLASH.^31,32^ Intense extreme ultraviolet (XUV) pulses at 91 eV photon energy were produced with an electron bunch charge of 0.5 nC, yielding a pulse energy of 150 *μ*J as measured with the gas monitor detector.^32^ An estimate for the pulse duration of 100 fs is derived for these parameters from measurements^33^ carried out with the electron bunch length diagnostics LOLA.^34,35^ Considering a beamline transmission of 64% and a focal spot size of 20 *μ*m (FWHM) at the beamline BL2,^32^ a power density of up to $3\times 10^{14}$ W/cm^2^ was reached. The pulses intersected a highly diluted jet of sub-micron xenon clusters.^36^ An adjustable piezo-skimmer slit ensured that only one single cluster was present in the focus volume per FEL shot.^15^ The scattering patterns were measured with a previously described^14,28^ large area scattering detector consisting of an MCP-phosphor stack with a center hole and an out-of-vacuum CCD camera. In addition to the diffraction images, coincident single-shot ion time-of-flight (tof) spectra were recorded.^37,38^ In the polarization plane of the FEL, the setup allowed us to measure diffraction patterns at scattering angles between 3° and 30°. In the perpendicular direction, the detection angle was limited to 10° due to a shadow of the spectrometer's electrodes. Prior to further analysis, the measured scattering intensities were corrected for a nonlinear detector response^9^ by taking each pixel's intensity to the power of 2.5 and for the flat detector geometry^28^ by multiplying with a factor of $\text{cos}{\left(\theta \right)}^{-3}$.

## III. RESULTS

**FIG. 1.**
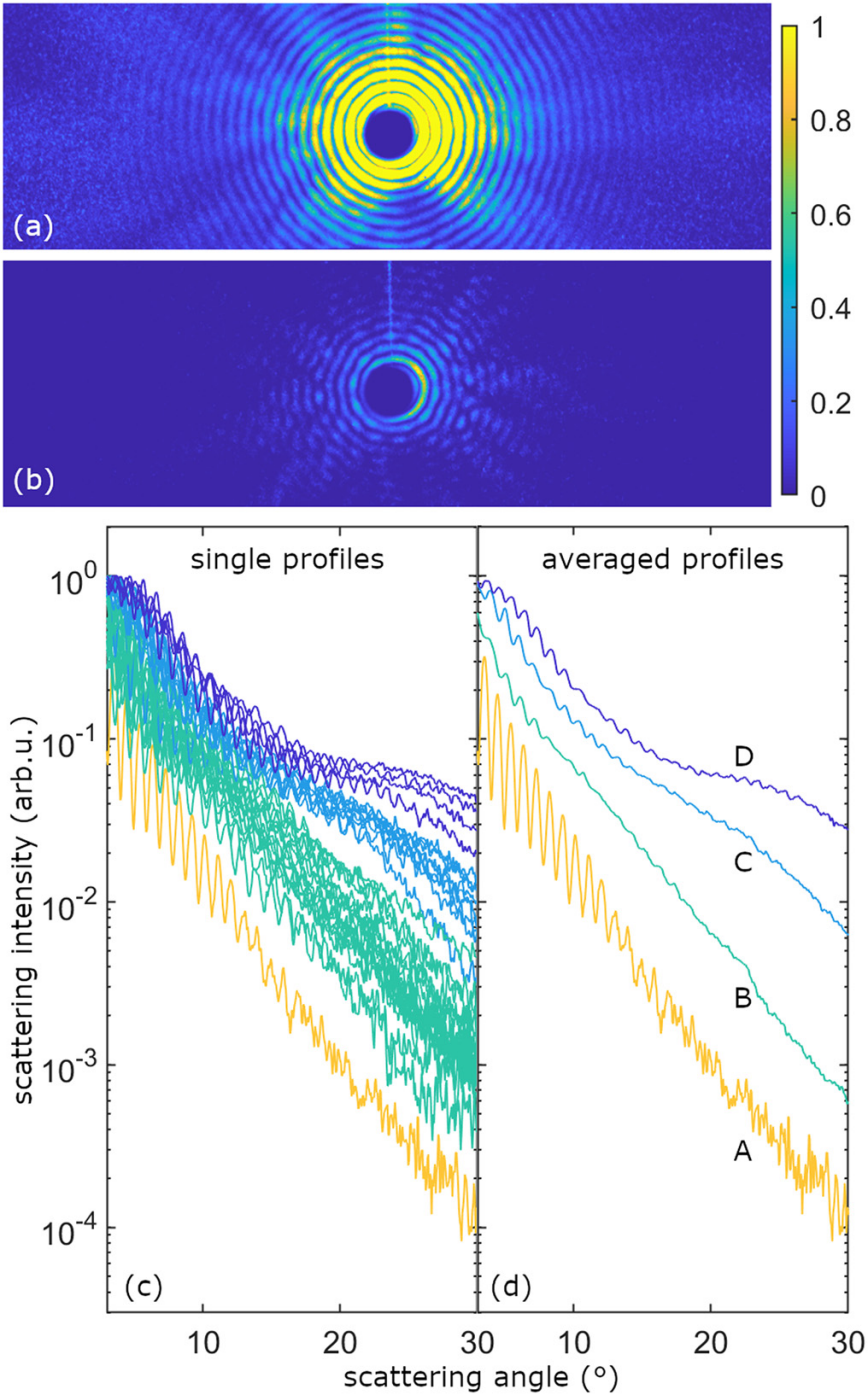
Isolated xenon clusters were irradiated with intense XUV pulses (91 eV photon energy and $3\times 10^{14}$ W/cm^2^ peak intensity in the center of the focal spot). A total of 32 events with single clusters of (400±50) nm radius were selected for analysis by the characteristic spacing of the diffraction rings. (a) and (b) Representative diffraction images (second brightest and darkest image of 32 events). (c) Radial profiles of the 32 single-shot images (corrected for the flat detector and nonlinear response, averaged over the scattering angle ϕ; see text). The color coding indicates the binning of events with similar intensities (the least intense category only contains a single pattern). (d) Radial profiles of averaged patterns from bins A to D. For increasing scattered intensity, an upward shift of the profiles (linear response) and an additional modulation of the profiles (corresponding to the ionization and plasma formation) can be observed.

Two representative examples of the single-shot single-particle diffraction patterns are shown in Figs. 1(a) and 1(b). The difference in brightness results from different irradiation intensities of the FEL due to the varying positions of the xenon clusters within the focal volume.^38^ As known from previous work, the basic structure of the diffraction images, with concentric but intermittent rings, indicates nearly spherical shapes with rough surfaces resulting from the coagulation-dominated growth process.^36^ The size of each single cluster (average radius) could be determined by comparing the spacing of the extrema in the diffraction patterns with Mie calculations.^36^ A total of 32 patterns within the size regime of R=(400 ± 50) nm were selected (the raw data of all patterns were uploaded to the CXIDB^39,40^). Then, the patterns were radially averaged and plotted on a logarithmic scale vs scattering angle in Fig. 1(c).

### A. Intensity dependent evolution

A number of observations can already be made when following the profiles' evolution in Fig. 1(c) from the lowest to the highest intensity: first, a high-frequency modulation can be observed in all profiles, originating from the ring structures in the patterns [see Figs. 1(a) and 1(b)] and reflecting the cluster size information. In the case of the least intense profile (yellow curve), the envelope agrees rather well with the expected curve for a homogeneous spherical xenon cluster, dropping linearly on a logarithmic scale. In the absence of light induced changes in the particle, all other profiles from the clusters irradiated with higher FEL intensity would follow a similar curve, just with a proportionally higher scattering signal. Instead, the envelopes of the more intense profiles show an additional large-scale structure.

In order to analyze only the intensity dependent signature in the patterns and to reduce effects from irregular shapes and slightly different sizes, the events were sorted for increasing detector brightness and binned into four different categories *A* to *D* [the bins are indicated by the color coding in Fig. 1(c)]. The diffraction patterns within each of the four bins *A* to *D* were averaged, and radial profiles were extracted from the averaged patterns. These averaged profiles are plotted in Fig. 1(d).

When following the averaged profiles of categories *A* to *D*, the evolution of the profile envelopes can be seen even more clearly. A lobe structure appears with a minimum roughly at a scattering angle of 15° that becomes more pronounced with increasing irradiation intensity. In general, a modulation in any diffracted intensity distribution corresponds to a characteristic length scale in the scattering object. We thus conclude that the evolving superstructure corresponds to the development of an additional scattering structure in the sample, an area with different optical properties. It is notable that the modulation structure appears to be a general feature because it survives the averaging over many single-cluster patters, which themselves incorporate the average scattering signal over the FEL pulse duration. This raises the question of the origin of the transient refractive structure with a spatial extension on the order of a few tens of nanometers, as estimated from an Airy pattern with a minimum at 15°.

The results from theoretical^41–53^ and experimental^14,20,37,38,54–67^ studies on clusters in intense XUV pulses provide basic knowledge about the interaction between an intense short wavelength pulse and a rare gas cluster. From this body of work, we can exclude ionic motion as the origin of the observed modulation feature. A general picture divides the dynamics in three phases.^42,48,54,68^ In the first phase, photoionization and Auger decay lead to the emission of electrons with residual kinetic energy from the cluster and therefore result in a charge-up of the cluster as a whole.^48,69^ As soon as the Coulomb attraction between the positively charged cluster and photoelectrons (or Auger electrons) exceeds their kinetic energy, the second phase starts, and subsequently, released electrons become trapped in the cluster potential.^41,56^ A nanoplasma is formed in which further electrons are released from the single atoms or ions, but they reside within the cluster. Within the following pico- to nanoseconds, in the third phase of the cluster dynamics, the electrons transfer their kinetic energy to the ions, expelling one surface layer after the other in a hydrodynamic expansion.^20,37,38,48^ Also, the net charge on the cluster leads to ion repulsion of the unshielded surface, referred to as Coulomb explosion.^45,57,60,70^ A theoretical study^48^ modeling argon clusters irradiated with 90 eV radiation predicts a motion of the outermost cluster shell of about 1.5 Å within the first 100 fs of the interaction for conditions comparable to the current experiment (the argon ions being a factor of 3 lighter and the intensity being a factor of 6 smaller).^48^ Furthermore, we can get a first-order estimate for the maximum motion of ions from the ion time-of-flight spectra measured in our experiment. An estimate for the acceleration at the surface can be derived from the final kinetic energies of the cluster ions of up to 600 eV per charge.^38^ Assuming a 400 nm sphere that accelerates a $Xe^{5+}$ ion to its final kinetic energy of 3000 eV yields an effective charge of that sphere of $10^{5}e$. Such a sphere drives $Xe^{5+}$, starting at the sphere's surface from rest, less than 1 Å within the first 100 fs.

Having excluded ionic motion, we attribute the observed modulations to light-induced electronic structure changes (i) resulting from the ionization and plasma formation within the FEL-irradiated particle and (ii) visualized by the use of a wavelength of the FEL resonant with neutral xenon and low charge states while being nonresonant with higher charge states. In Sec. III B, based on a first-order model of the cluster ionization process, we develop a physical picture of the plasma formation and discuss the generation of an outer shell in the cluster with strongly altered optical properties. Subsequently, using a classical concentric core–shell Mie model, we fit the modulation features observed in Fig. 1 in order to extract tendencies for the evolution of the shell’s thickness and optical constants with increasing irradiation intensity (Sec. III C).

### B. Simulation of the charge state distributions

**FIG. 2.**
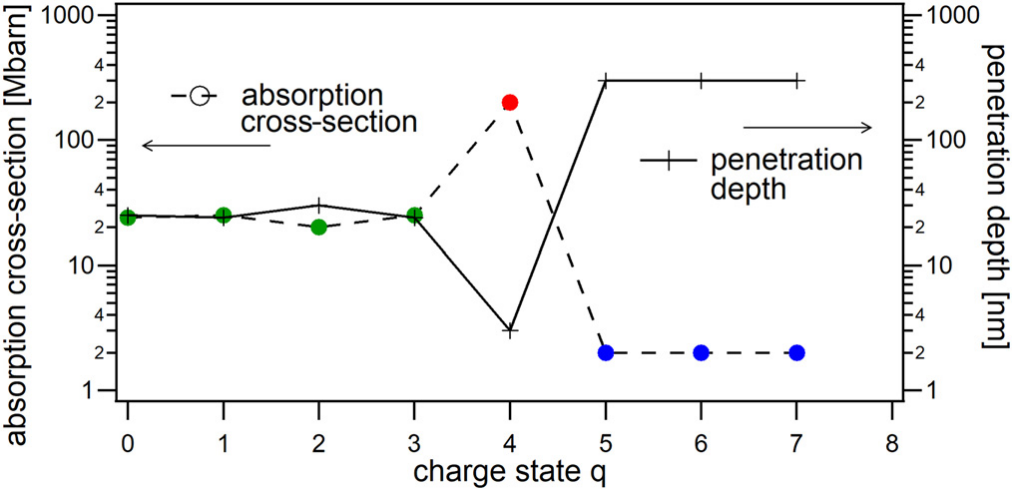
Absorption of neutral xenon atoms and atomic ions at 91 eV. Total absorption cross sections *σ_abs_* in Mbarn of neutral Xe,^71^ Xe^+^,^72^ $Xe^{2+}$,^73^ $Xe^{3+}$,^74^ $Xe^{4+}$,^75^ and $Xe^{5,6,7+}$^76^ (colored points). Note that the value of 2 Mbarn for 5 to 7+ constitutes an upper bound. The corresponding penetration depth in nm (black crosses) is calculated using $l_{\mathrm{abs}}=\frac{1}{n_{a}\cdot \sigma_{\mathrm{abs}}}$, with *n_a_* being the atomic density of solid xenon.^77,78^

The appearance of the dynamic scattering features can be connected to the peculiar electronic properties of xenon atoms and ions in the vicinity of the photon energy of 91 eV. Absorption cross sections for gas-phase xenon atoms and atomic ions have been measured^71–76^ and are summarized in Fig. 2. A clear step from high to low absorption between $Xe^{4+}$ and $Xe^{5+}$ can be observed, with extremely high values for the charge state 4+, which exhibits a large ionic resonance at 91 eV. Correspondingly, the penetration depth, i.e., the depth into the material for the intensity to decay to 1/e, increases from about 30 to 300 nm.

**FIG. 3.**
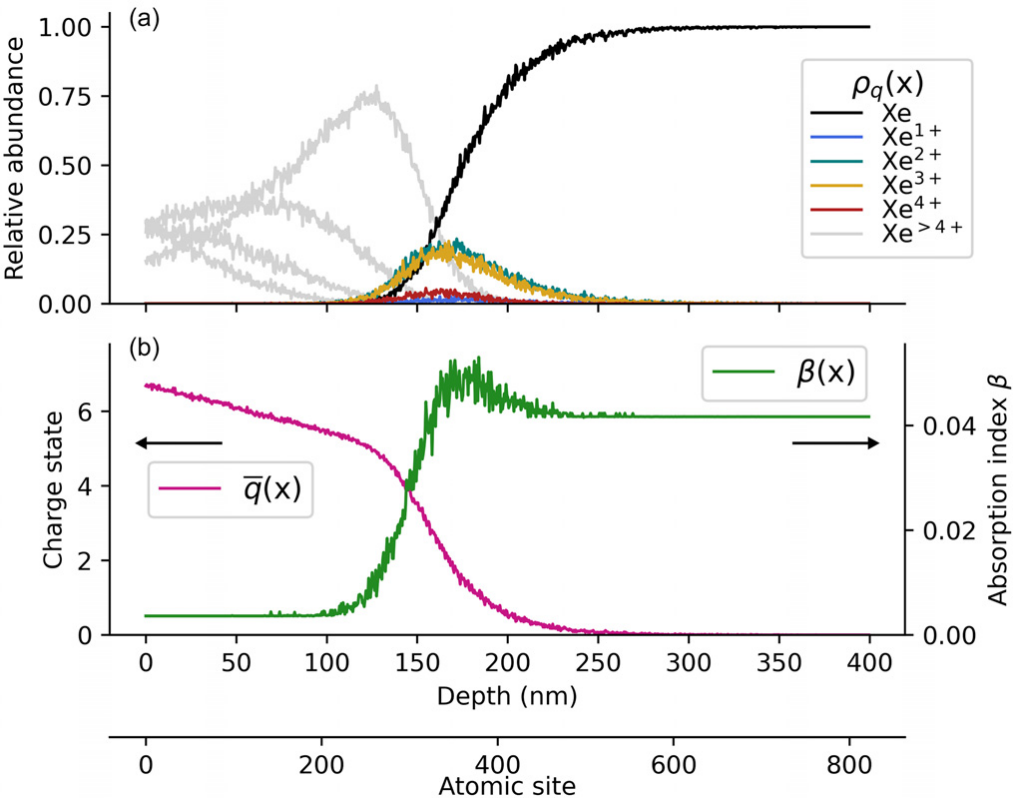
(a) Simulation of the distributions of the relative charge state abundances $\rho_{q}(x)$ for a one-dimensional chain of 824 atoms, i.e., 400 nm length. 870 photons (corresponding to $10^{14}\;W/cm^{2}$) fall on the geometric cross section of one xenon atom and are propagated along the chain. Absorption cross sections of atomic xenon and its ions from Fig. 2 are used for calculating absorption probabilities. (b) The average charge state $\bar{q}(x)$ drops from around 6+ to neutral within 80 nm. The relative charge state abundances further allow us to determine an effective absorption index β(x), revealing a transition within 50 nm by an order of magnitude.

To gain a first-order model of the radial distributions of different charge states in an irradiated cluster, we investigate the photoionization of a one-dimensional chain of atoms using an atomistic Monte Carlo approach. The results are presented in Fig. 3. For the simulation, each photon is “propagated” along the chain, starting at *x *=* *0 from the first atom, by probing at every atomic position whether an absorption is taking place. If the random drawing dictates an absorption process, the photon is annihilated, and the charge state of the corresponding atom is increased by 1 (or 2 in the case of an Auger process), followed by the start of the propagation of the next photon, again at *x *=* *0. According to the number density of solid xenon^77^ of $n_{a}=1.67\times 10^{28}$ particles per m^3^, we consider 824 atoms for a chain length of 400 nm. The charge state dependent absorption probabilities for each atom are derived from the absorption cross sections of different xenon charge states.^71–76^ Only linear photoabsorption and subsequent Auger processes are taken into account, while nonlinear effects, light scattering, and plasma processes such as collisional ionization are neglected. The derivation of the absorption probabilities and the number of photons impinging on the one-dimensional chain of atoms, as well as a benchmarking of the model by simulating the penetration depth for individual charge states, are given in the supplementary material.

In Fig. 3(a), the simulated distributions of the relative charge state abundances $\rho_{q}(x)$ with *q *=* *0 to 8+ are presented for an irradiation intensity of 10^14^ W/cm^2^, corresponding to 870 photons falling on the respective area of a single xenon atom (calculated via $\pi \cdot r_{Xe}^{2}$ with the atomic radius of xenon *r_Xe_* being determined from the solid density value^77^ given above). From the relative charge state abundances, the distribution of the average charge state $\bar{q}(x)$ and an effective imaginary part of the refractive index β(x) along the chain are calculated,^78^ as given in Fig. 3(b). Note that the complex refractive index n=1−δ+iβ is a dimensionless quantity with the absorption index *β* and the so-called refractive index decrement *δ*, which is related to the phase shift of light traveling through matter. The effective absorption index along the chain is derived as

$$\beta (x)=\sum_{q=0}^{8}\rho_{q}(r)\cdot \beta_{q}\;\;\text{with}\;\beta_{q}=\frac{1}{4\pi }\lambda \cdot n_{a}\cdot \sigma_{\mathrm{abs},q},$$

using the atomic/ionic cross sections given in Fig. 2 and the wavelength *λ*. Both curves given in Fig. 3(b) indicate that after irradiation, an outer shell exists up to a propagation depth of about 120 nm. In this depth, the average charge state $\bar{q}(r)$ drastically drops in a transition region of about 80 nm thickness from about six to zero, while the effective absorption index β(r) jumps from 0.004 to 0.05, revealing an even more pronounced kink.

Our basic one-dimensional Monte Carlo simulation therefore indicates the formation of an outer shell in the cluster with only highly charged ions, which is rather transparent as compared to the opaque core. As mentioned above, the simulation omits nonlinear effects, the light scattering process itself, and all plasma related processes such as collisional ionization. While it yields simulated values of the imaginary part of the refractive index *β*, it does not allow for a prediction of possible changes in the refraction (*δ*) of the shell. Nevertheless, it provides a first-order explanation for the dynamic diffraction feature arising from the resonant interaction of 91 eV radiation with the xenon clusters.

### C. Core–shell Mie fitting

The ionization model described above indicates the formation of an outer part in the cluster with altered optical properties, but it is important to note that the geometrical nanoplasma structure may considerably deviate from a *concentric* core–shell system. Instead, the considerations of the ionization process suggest a distribution that is asymmetric in the direction of the incident light, with a transparent part at the irradiated side of the cluster, while the back of the cluster remains neutral. However, assuming a concentric core–shell offers the advantage that the patterns can be further analyzed using classical core–shell Mie theory,^79,80^ i.e., the analytic solution of the Maxwell equations for the case of a concentric core–shell system. The input values and parameters of the Mie simulation—the size of particle and shell, the complex refractive index in both areas, and the wavelength and intensity of the incoming light—provide important handles to capture main tendencies of the nanoplasma formation. While the influence of the expected asymmetry needs to be tested with advanced theoretical models,^70^ we note that the contribution from the back of the cluster to the diffraction should be also small in a concentric Mie-model with a highly absorbing core, shadowing the back of the cluster from irradiation.

For the Mie analysis, not the averaged patterns *A* to *D* obtained at similar irradiation intensities [radial profiles are shown in Fig. 1(d)] are considered but their difference signal. This approach is conceptually similar to resonant x-ray imaging, e.g., of the ultrafast switching of magnetic domains,^81^ where the difference signal between patterns obtained at different helicities of circularly polarized light is analyzed, or of buried structures,^30^ where diffraction patterns just above and below an absorption edge are subtracted from each other to enhance the difference signal in the location of a certain element.

Using a difference signal approach in combination with Mie fitting is based on the following considerations: the measured patterns (and also averaged data) do not correspond to a single, intermediate plasma state that can be approximated by a single nanoplasma structure such as a single core–shell system. Always, the onset of the FEL pulse intercepts a cluster that is neutral and unchanged, and the last photons of the pulse interact with an evolved nanoplasma. By analyzing the difference spectra with Mie core–shell fits, we discretize this evolution and link the intensity-resolved information in different profiles with the idea of a common plasma dynamics (see also the mathematical derivation in the supplementary material).

This includes the simplifying assumption that the patterns belonging to groups *A* to *D*, obtained at different irradiation intensities, all result in principle from the same continuous evolution of nanoparticle ionization and plasma formation but up to different stages. In other words, profile *A* corresponds to an only weakly irradiated cluster, profile *B* contains the response of this initial phase and additionally the response of a more advanced nanoplasma, and so on. This perspective is equivalent to the statement that the number of impinged photons is the decisive factor for the plasma state reached. This assumption allows us to replace the variable of time by the variable of energy, but it can only be a rough approximation. Implicitly, this means that all nonlinear processes such as multiphoton absorption are neglected (for the instantaneous absorption of two photons, the intensity, i.e., the number of photons per time interval, is relevant, not only the number of photons in total).

**FIG. 4.**
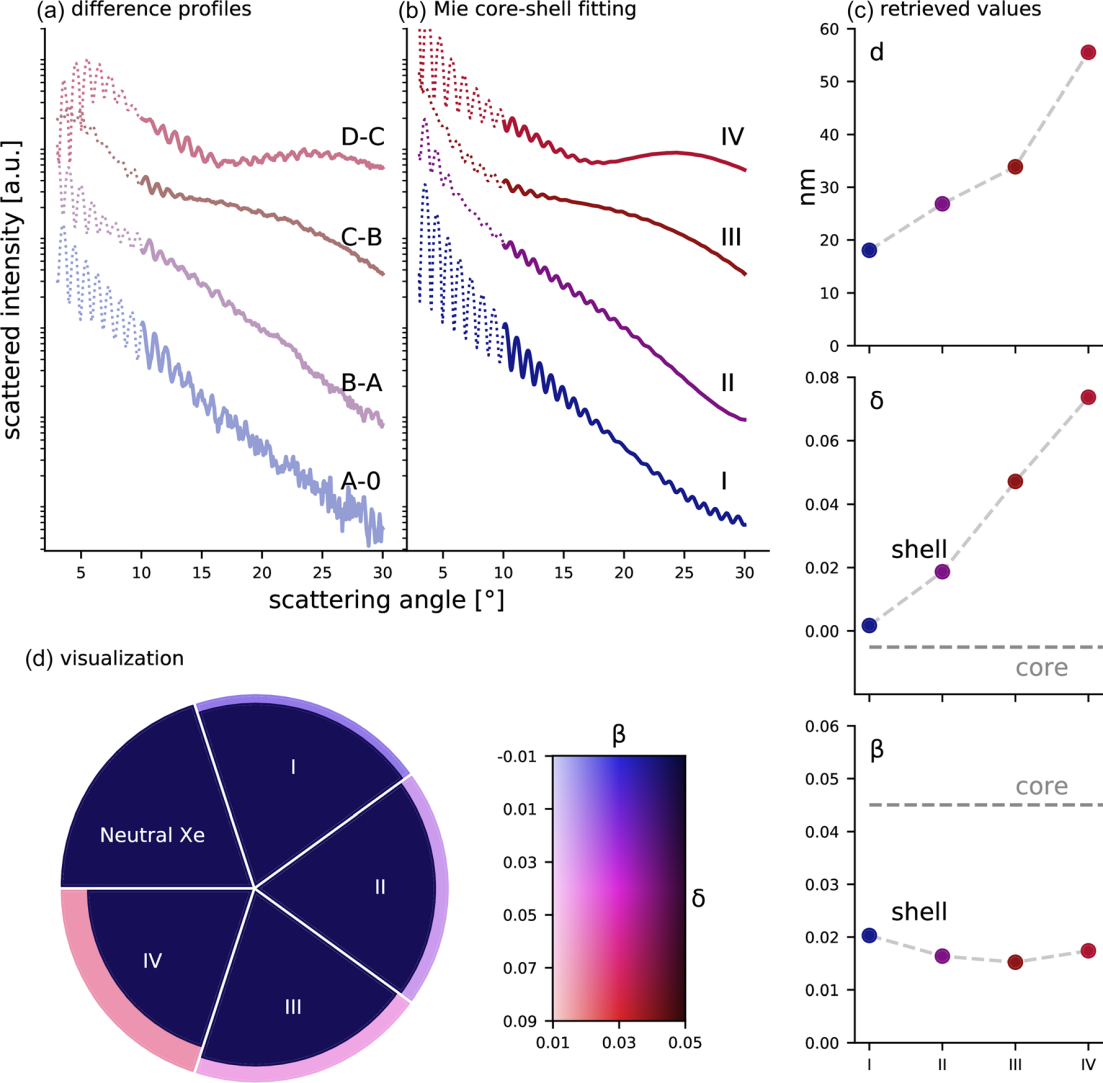
(a) Difference profiles from the averaged profiles shown in Fig. 1(d). For better visibility, the upper curves were shifted by multiplication with a factor. (b) Fitted core–shell Mie profiles using the code from Shen.^82^ The refractive index of the core was kept constant to n=1.004+**i**·0.045 [values of neutral xenon at 91 eV (Ref. 84)]. See text for details. Analog to (a), the profiles II–IV were shifted by a multiplicative factor for better visibility. Dashed lines in (a) and (b) show the profiles below an angle of 10°, where the experimental data were excluded from the fitting process. (c) Parameters of the shell obtained from the fitting, i.e., shell thickness *d* (in nm), absorption index *β*, and refractive index decrement *δ*. (d) Visualization of the sequence of core–shell structures derived from the fitting with changing parameters of the shell [for the exact values of the refractive indices, compare with the 2D color map or with the graphs for *β* and *δ* given in (c)].

Now, instead of a gradually evolving and changing system, we may approximate the nanoplasma formation process by a sequence of a few discrete steps. The profiles of the difference signals *D*–*C*, *C*–*B*, and so on are fitted with a concentric core–shell Mie model (note that profile *A* can be seen as the difference between *A* and 0). In Fig. 4(a), the radial profiles from the difference signal of the averaged patterns *A* − 0, *B* − *A*, *C* − *B*, and *D* − *C* are given. The difference profiles reveal even more distinct features in the superstructure: with increasing irradiation intensity, a broad lobe appears, which becomes more and more pronounced, narrows, and shifts toward higher scattering angles.

With a Mie code that was extended for spheres with a core–shell structure,^79,80,82^ profiles were simulated and fitted to the experimental curves [see Fig. 4(b)]. A global optimization was carried out using a differential genetic algorithm.^83^ This approach, even if it is among the slowest optimization methods, has the advantage of being flexible, and it does not require an initial guess for the solution. The scattering angle in a range between 10° and 30° was selected for the fitting because smaller angles are prone to detector saturation effects [see single profiles in Fig. 1(c)]. The optimization target was set to the L2-norm of the logarithm of the profiles (i.e., least squares minimization on the logarithmic scale). The shell thickness *d*, the refraction decrement *δ_shell_*, and the absorption index *β_shell_* were varied within the parameter bounds given in Table I. For the core, the values of neutral xenon at 91 eV photon energy^84^ were used (see Table I). It is noted that the fitting results were rather insensitive to the choice of the core's refractive index, as long as the absorption of the core was considerably higher than that of the shell. Instead of using a single particle size, the average profiles from several spheres with different sizes, following a distribution given by the scaling parameter *σ*, were fitted to the experimental profiles. This allows us to make the fitting insensitive to influences from the high-frequency oscillations of the profiles corresponding to the cluster size by taking into account the individual particles' deviation from spherical shape and the difference in sizes. For further details and observations on the fitting procedure, see the supplementary material.

**TABLE I.** Fixed values and parameter bounds of the input parameters for the fitting routine. The literature values of neutral solid xenon at 91 eV are taken from Henke tables.^84^

Literature values	$\delta_{\text{Xe},91\text{eV}}$	−0.004
	$\beta_{\text{Xe},91\text{eV}}$	0.045
Parameter bounds	$d_{\text{shell}}$	(0, 100) (nm)
	$\delta_{\text{shell}}$	(−0.1, 0.1)
	$\beta_{\text{shell}}$	(0, 0.1)
	$\sigma_{\text{shell}}$	(0, 50) (nm)

The parameters of the shell resulting from the global optimization are given in Fig. 4(c). We find (i) a compared to the core low value of *β* around 0.02, which indicates a transparent shell, (ii) an increasing shell thickness of 20–55 nm, and (iii) a refractive index decrement *δ* increasing with intensity with positive values up to 0.08. The same fitting results are obtained for a broad range of starting values (cf. Table I). Systematic Mie simulations, in which the three parameters are varied separately, are shown in the supplementary material. They support the assumption of a single optimal parameter set, as they show that the three parameters *d*, *β*, and *δ* have a separable influence on different characteristics of the core–shell signature. The fitting results also provide a scaling factor for the incoming number of photons and thus enable an estimate of the incoming intensities for the average profiles *A* to *D* in Fig. 1(d). Assigning the maximum irradiation intensity, $I_{D}=3\times 10^{14}$ W/cm^2^ to group *D*, the values for the other average profiles can be determined according to $I_{A}=6\times 10^{13}$ W/cm^2^, $I_{B}=1\times 10^{14}$ W/cm^2^, and $I_{C}=2\times 10^{14}$ W/cm^2^.

## IV. DISCUSSION

Our one-dimensional first-order ionization model indicates that the transient characteristic length, revealed by the observed modulations, is connected to the formation and evolution of a highly charged outer shell with dramatically changed optical properties. The fact that we are able to fit the experimental profiles well with a sequence of concentric core–shell Mie models further supports the general physical picture, even though both models are clearly limited. For gaining a clearer picture, a full description of the light propagation via sophisticated many-particle simulations will be needed, which includes also other processes such as impact ionization, charge transfer, plasma shifts of the energy levels, and further nanoplasma dynamics.^85–87^ Nevertheless, our general considerations provide a first step toward understanding the observed results.

The results from the Mie-calculations are in good qualitative agreement with the atomistic ionization model. They show the same general trend of a rather transparent outer shell, whose thickness increases with the irradiation level. From the Mie-fitting, we further extract a tendency for the real part of the refractive index, indicating a strong change in the refraction between the core and the shell, which grows with the incoming intensity. This general agreement in combination with the robustness of the fitting results indicates that key trends are captured in the analysis.

On the other hand, the large differences in the absolute values of shell thickness and absorption index demonstrate the limitations of our modeling approaches. In addition, while the Mie approach assumes a discontinuous transition between the core and the shell, the ionization model indicates a transition range on the order of 50 nm thickness. This may partly explain the higher absorption values in the Mie fits since a higher absorption can in principle take over the effect of a gradual transition (see the variation of beta in the supplementary material). Nevertheless, a transition region of such broadness cannot actually explain the formation of an observable scattering feature. We have tested with a simple scattering simulation (see the supplementary material) that the modulation in the scattering profiles disappears already for a transition region of half that thickness. The required sharp transition is puzzling and cannot be explained by our simple models.

The following considerations may allow us to hypothesize on the origin of a narrow reflective layer tens of nanometers deep in the nanoplasma. In general, a high reflectivity is connected to a strong change in the *real part* of the refractive index. The above discussed first-order ionization simulation only describes the absorption of the nanoplasma, i.e., the imaginary part *β*, and a model of its radial dependence. For the optical response of the cluster, both the real and imaginary parts of the refractive index, 1−δ and *β*, are relevant, which are interrelated through the Kramers–Kronig dispersion relations. In this context, the optical properties of the charge state $Xe^{3+}$ are worth a closer look. Plasma calculations of the atomic scattering factors of $Xe^{3+}$ indicate that between 90 eV and 98 eV, the real part of the atomic scattering factor *f*_1_ (proportional to the refractive index decrement *δ*) rapidly changes from strongly positive to negative values and back several times.^88^ Now, we have to take into account that in the environment of the nanoplasma, the $Xe^{3+}$ ions are not isolated (as in the gas-phase measurements carried out to determine the atomic absorption cross sections^74^) but instead surrounded by other ionic species and quasi-free electrons in the nanoplasma. By comparing the $Xe^{3+}$-distribution [orange curve in Fig. 3(a)], peaking between 150 and 200 nm in depth with the average charge state at the same x-positions [magenta curve in Fig. 3(b)], we find that the neighborhood of the $Xe^{3+}$ ions drastically changes. In a plasma environment, atomic or ionic resonances can be shifted in energy up to several eV.^89^ It is to be expected that the change in the environment as a function of the propagation depth translates into an energy shift, possibly from just below a sharp resonance to just above the resonance. This would result in a drastic change of the real part of the refractive index within a short distance, acting like a transient plasma mirror. A similar argument could be made for other xenon charge states $Xe^{4+}$ to $Xe^{6+}$, which also exhibit narrow and very strong absorption resonances in the vicinity of 91 eV.^74,75^ For testing this hypothesis, more sophisticated theoretical approaches will be required.

## V. CONCLUSION

In summary, we have presented scattering patterns of single large xenon clusters resonantly excited with intense XUV pulses. The patterns reveal strong intensity dependent modulations in the radial distribution of the diffracted light, indicating the formation of an additional characteristic scattering length scale in an otherwise homogeneous nanoscale particle. Using a first-order modeling of the ionization in combination with Mie-based simulations, we assigned the transient diffraction signal to light induced electronic core–shell structures with an increasingly thick outer shell of low absorption and high refraction. An abrupt change in refraction, needed to explain the prominent diffraction feature observed, may be correlated with the radially changing plasma environment of higher charge states, translating into a radially changing shift of the electronic resonances. Our work shows that ultrafast resonant light scattering can map the transient spatial charge distributions in laser-excited nanoscale matter. The method can be employed to develop a deeper understanding of nanoplasma formation and charge transfer dynamics, which play a key role in many areas ranging from single-shot x-ray imaging to fusion and warm dense matter research and condensed matter physics. In the future, the approach provides an avenue to resolve ultrafast electron dynamics in extended systems on their natural timescale with intense attosecond pulses currently under development at FELs and lab-based sources.^90–95^

## SUPPLEMENTARY MATERIAL

See the supplementary material for a mathematical expression of the discretization approach, further details on the fitting procedure, a systematic investigation of the influence of each input parameter on the modulation features, simulations for sharp and smooth interfaces, and the derivation of the radial absorption index.

## Data Availability

The data that support the findings of this study are openly available at the CXI data bank at http://cxidb.org/id-146.html (reference number, 146).
